# Notes on the Pneumogastric

**Published:** 1873-10

**Authors:** J. F. Alleyne Adams

**Affiliations:** Pittsfield


*Notes on the Pneumogastric.* By J. F. ALLEYNE ADAMS, M.D., Pittsfield. Read before the Massachusetts Medical Society, June 3, 1873.

In presenting to the Society a brief paper upon the functions of the pneumogastric nerve, and their bearing upon the etiology and treatment of certain forms of disease, a few remarks will be offered, by way of introduction, upon the anatomical relations of the pneumogastric to the other cranial nerves.

By the classification of Soemmering, the pneumogastrics constitute the *tenth* pair, and by that of Willis a portion of the *eighth.* The more scientific classification of Muller calls the pneumogastric, with the glosso-pharyngeal and spinal accessory, the *second* pair of cranial nerves, the third, fourth, fifth and sixth of Willis, with the portio dura of the seventh, being grouped together to form the first, while the hypoglossal is the third. This arrangement is more in accordance with the teachings of philosophical anatomy, inasmuch as it harmonizes the nerves with the vertebrate type of the skull, giving one pair for each of the three cranial segments. It leaves out of the question the three nerves of special sense, viz., olfactory, optic and auditory, which are demonstrated by Oken to have no true nerve structure, but to be mere outgrowths or appendages of the brain. But Muller's classification does not complete the homology between the cranial and spinal nerves, for the third pair has no sensitive root, and the distribution of both the second and third is greatly at variance with the anatomical relations of the vertebræ to which they respectively belong.

Dalton* proposes a fourth classification, which is identical with Muller's, excepting that the hypoglossal is put with the glosso-pharyngeal to form the second, while the pneumogastric and spinal accessory constitute the third. Although suggested by him on purely physiological grounds, this arrangement commends itself, as well, from an anatomical point of view; and there are strong arguments in favor of its adoption as the true classification of the cranial nerves.

* Dalton's Human Physiology, p. 432.

These arguments may, for convenience, be arranged under the three following heads:

1st, Origin; 2nd, Distribution; 3rd, Function.

1st. *Origin.* All of the cranial nerves, excepting the three nerves of special sense mentioned above, have their deep origin in the medulla oblongata, and are arranged in the following order: from its upper portion arise, in close proximity, the trigeminus, the facial, motor oculi, abducens and patheticus; then comes the glosso-pharyngeal, next below which is the deep origin of the hypoglossus; below this is the vagus, and, lastly, the accessorius. Thus we see that the hypoglossus, although having its origin on the same level as the vagus, and anterior to it, really rises above that nerve, and close to the origin of the glosso-pharyngeus. Arguing from the deep origin, therefore, there is no propriety in calling the hypoglossus the last of the cranial nerves.

2nd. *Distribution.* The several nerves grouped together as the first pair are distributed chiefly to the frontal segment, including its inferior or hæmal arch, the inferior maxilla. The second, or parietal segment, has for its hæmal arch the styloid processes and hyoid bone; and we find the glosso-pharyngeus and hypoglossus distributed to the tongue and pharynx, parts intimately connected with these bones. The third, or occipital segment, by an adaptation to special ends, has its hæmal arch separated from it, to form the scapular arch; and, accordingly, the third pair of nerves undergoes a similar deviation, the pneumogastric and accessorius being distributed to the whole respiratory apparatus, the heart and chylo-poetic viscera, while the spinal portion of the spinal accessory goes to the trapezius and sterno-mastoid muscles, which form the chief connecting links between the occipital vertebra and its hæmal arch.

3rd. *Function.* The present classification makes the analogy of the cranial to the spinal nerves the more striking, in that it gives to each pair a sensitive and a motor root. Thus, the first pair has for its sensitive root the trigeminus, and for its motor the facial, of which the three motor nerves of the eye and the motor root of the fifth pair are mere accessories. The second pair has the glosso-pharyngeal for its sensitive, and the hypoglossus for its motor root, while the sensitive root of the third pair is the vagus, and the motor the spinal accessory.

Of the arguments that may be adduced in antagonism to this classification, the strongest is, that, in frogs, the hypoglossus is placed *below* the vagus, and becomes the first of the spinal nerves; although the deep origins of the two nerves, even in this case, have been shown by Vogt to be close together.* Another objection is, that the hypoglossus leaves the skull by the anterior condyloid foramen, which is posterior to the foramen lacerum posterius,

* J. Wyman. Nervous System of Rana Pipiens. Washington, 1853.

which transmits the pneumogastric. Therefore, although both of these objections might possibly be ruled out, the arrangement is not to be accepted without due caution.

The pneumogastric arises, superficially, by eight or ten filaments, from the lateral portion of the medulla oblongata, behind the olivary body, between the roots of the glosso-pharyngeal and accessorius. Its deep origin is from a grey nucleus in the lower portion of the floor of the fourth ventricle. The nerve leaves the cranial cavity by the foramen lacerum posterius, within which is formed the ganglion of the root, the ganglion of the trunk being external to the cranium. The root of the pneumogastric is strictly sensitive, but its trunk is both sensitive and motor. Its motor filaments are derived chiefly from the accessorius, which unites with it just below the ganglion of the trunk, besides giving off some filaments to the ganglion of the root. It likewise anastomoses at the ganglion of the root with the facial and hypoglossal, and, at that of the trunk, with the hypoglossal and first two cervical nerves. Both ganglia are intimately connected by numerous filaments with the superior cervical ganglia of the sympathetic, while its trunk receives branches from the middle and inferior cervical and upper dorsal ganglia.

Thus constituted and reinforced at its origin, the par vagum is prepared for a distribution more extensive and functions more varied and remarkable than any other pair of nerves. Its functions may be briefly stated as follows:—Its *sensitive* filaments are distributed, through the auricular, to the external auditory meatus and membrana tympani, through the superior and inferior laryngeal, anterior and posterior pulmonary, to the whole respiratory tract, through the cervical and thoracic cardiac to the heart, and through the pharyngeal, œsophageal and abdominal branches to the digestive tract.

Its *motor* filaments are distributed, through the pharyngeal and inferior laryngeal branches to the pharynx, through the recurrent laryngeal to all of the muscles of the larynx except the crico-thyroid, through the superior laryngeal to the crico-thyroid, through the recurrent laryngeal and pulmonary branches to the tracheal and bronchial muscular coats; and, through the œsophageal, gastric and intestinal branches to the muscular coats of the œsophagus, stomach, and the whole of the small intestine. Until the discovery of the intestinal branches by Kollman, in 1860, the abdominal distribution of the vagus was supposed to stop at the pylorus.

The pneumogastric sends *secretory* fibres to the gastric and intestinal mucous membrane.

Its hepatic branches preside over the glycogenic function of the liver.

It also sends inhibitory or arresting fibres to the heart.

It transmits accelerating and inhibiting fibres to the centre of respiration.

It sends accelerating and inhibitory fibres to the vaso-motor centre, the latter being contained in the depressor nerve of Cym and Ludwig.

Without attempting to go over all of the ground here indicated, this paper will confine itself to the more recent discoveries concerning the relations of the pneumogastric to the heart, the lungs, and the stomach.

*Influence of the Pneumogastric upon the Heart.*—Although the vagus supplies to the heart both centripetal and centrifugal filaments, its action upon this organ is not that of an ordinary motor nerve. On the contrary, it exerts an arresting or regulating influence. The rhythmical contractions of the heart are induced by an excitation derived from the cardiac ganglia of the sympathetic system, beautifully described and figured by Pettigrew, in the May number of the *Edinburgh Medical Journal* for the present year.* This system bears to the heart the same relation that the main-spring does to a watch, while the pneumogastric answers, in some sort, to the escapement. This influence is commonly known as the *inhibitory* action of the pneumogastric.

The subject of inhibition was first introduced to the profession in 1846, by the brothers Weber, who discovered that galvanization of the pneumogastrics in the neck rendered the action of the heart slow; and if the current were sufficiently powerful, arrested the heart in diastole. Since then, numerous experiments have demonstrated the accuracy of their observations, and have added largely to our knowledge in this direction. The result of these experiments may be stated as follows: †

If one of the pneumogastrics be divided in the neck, the frequency of the heart's pulsations is slightly increased and the cardiac pressure slightly diminished.

If both pneumogastrics are divided, the frequency of the pulsations is greatly increased, at least doubled, while the force is greatly diminished.

Galvanization of the peripheral end of the divided nerve retards or arrests the heart's action; but galvanization of the central ends produces no such effect, showing that the inhibitory influence is a centrifugal one.

If an animal be placed under the influence of woorara poison, which paralyzes motor nerves without affecting sensitive nerves or muscular irritability, and the pulsations of the heart be kept up by artificial respiration, galvanization of the pneumogastrics has no

* James Bell Pettigrew. On the Physiology of the Circulation. Edinburgh Medical Journal, January to May, 1873.

† Flint. The Physiology of Man. Vol. IV. Nervous System. 1872.

effect upon the heart's pulsations; whence we may conclude that the inhibitory power is located in motor filaments. This experiment has been tried by Bernard and verified by Flint.

The experiment has been performed by Traübe of injecting digitalis into the veins of a dog with the pneumogastrics divided, without affecting the heart's pulsations; although, when this was done with the nerves intact, the number of beats was, in an hour, reduced to one-fourth of the normal number. This indicates that the sedative action of digitalis upon the heart is due to an irritation of the vagus, transmitted from the brain.

Irritation of the medulla oblongata produces the inhibitory action; but the irritation is conveyed, not by the root of the vagus, but, as has been demonstrated by Waller, by the accessorius.

Recently, it has been discovered that the right vagus possesses a greater inhibitory power than the left. "Dr. Massin states that, while the movements of the heart are stopped by a very powerful excitation of the left vagus, it is always possible, by sharply striking the heart with the finger, to reproduce the rhythmical movements; while this mechanical cause of action remains ineffectual when applied to the heart stopped by strong galvanization of the right vagus."*

The inhibitory influence upon the heart is also exerted in various reflex ways. Brown-Sequard† has found, in rabbits and guinea pigs, that an irritation of the nostrils by carbonic acid or chloroform, or even by a sudden and gentle touch, will cause the heart to stop completely, or, at least, notably diminish its force and speed. Rutherford states that, if any irritating vapor be brought before the nose of a rabbit, it closes its nostrils and ceases to breathe, often for thirty or forty seconds. Within three seconds after the cessation of respiration, the heart comes almost to a stand-still, and continues to beat very slowly until respiration is re-established. Rutherford believes that "this arrest of the heart is due to stimulation of the inferior cardiac branch of the vagus by the asphyxiated condition of the blood;" but Brown-Sequard proves conclusively that this is not the case, but that the phenomenon is due to reflex influence.

The cold bath, especially the shower baths or douche, has, by reflex action, the same inhibitory influence upon the heart. Fainting is even produced in some persons by this means, from complete arrest of the pulsations. Brown-Sequard states that, in Europe, where ice-water is not as habitually used as here, a stoppage of the heart's action is not rare, in the summer season, after the drinking of a large quantity of cold water.

* Brown-Sequard. On the Sudden or Rapid Arrest of Certain Normal or Morbid Phenomena. Archives of Scientific and Practical Med., Jan., 1873.

† Ibid. Most of the physiological facts in this paper are from Flint or Brown-Sequard.

Wounds of the abdominal viscera and blows upon the belly produce fainting or slowing of the heart's action. To Brown-Sequard we are indebted for the discovery that the crushing of the ganglions of the sympathetic in the abdomen stops or diminishes the movements of the heart; but if the spinal cord or vagus is divided transversely, the crushing can be made without any influence upon the heart.

Goltz has demonstrated, in the frog, that the heart can be brought to a stand-still by striking upon the abdomen, and that this paralysis of the heart can be prevented by a simultaneous strong irritation of the cutaneous nerves, which diminishes or abolishes the reflex activity.

It is asserted by Rouget, that the inhibitive action takes place in nerve-cells. He says: "Wherever the excitation of a nerve is followed by an arrest of movement, nerve-cells are found on the nerve-fibres which transmit the excitation." In this view he is supported by Brown-Sequard, who thus describes the process of arrest:

1. An irritation starts from a part of the nervous system radiating towards the nerve-cells, the activity of which is to be arrested.

2. A special influence is to be exerted by this excitation on those cells, arresting their activity.

3. The movements, normal or morbid, or other phenomena depending on the activity of those cells, cease at once when that activity ceases.

In the discovery of the *depressor* nerve by Cym and Ludwig, who first published an account of it in 1867, an important addition is made to our knowledge of the pneumogastric in its relation to the heart. This nerve was found in the rabbit, arising by two roots, one from the trunk of the pneumogastric, and the other from its superior laryngeal branch, passing down the neck, by the side of the sympathetic, to the chest, where, joining with sympathetic filaments, it passes to the heart by little branches between the origin of the aorta and the pulmonary artery. This nerve has since been found by Cym in the horse. In the rabbit it has been made the subject of a very complete and satisfactory series of experiments.

When this nerve is divided in the neck, and its *peripheral* end irritated by galvanization, no effect whatever is produced upon the heart or the circulation; but if its *central* end is galvanized, the pressure is diminished in the whole arterial system. This diminution is gradual, the pressure never reaching its minimum before fifteen pulsations of the heart, and may be reduced to one-half the pressure before the irritation was applied. At the same time that the arterial pressure is diminished, the frequency of the heart's pulsations is reduced, *unless both pneumogastrics have previously been*

*divided*, when, although the arterial pressure is reduced as before, the action of the heart is not disturbed. These experiments prove that the action of the depressor is entirely centripetal, that its inhibitory action upon the heart is reflex, through the nerve-centres, the trunk of the pneumogastric, and its remaining cardiac branches, while the reduction in the pressure of blood in the large arteries is independent of the centrifugal fibres of the vagus, and bears no relation to the reduction in the number of cardiac pulsations.

In determining the mechanism whereby the reduction in the arterial pressure is effected, Cym has displayed great ingenuity. Having, by a process of exclusion, eliminated all other possible agencies, he has clearly shown that this influence is exerted by reflex action, through the instrumentality of the splanchnic nerves, the most important vaso-motor nerves in the entire organism. "The irritation of the depressor nerve, after section of the splanchnic nerve," to use the language of Cym, "produced still a diminution in the blood-pressure, but the absolute value of this diminution is much less than it was during the irritation of the depressor nerve, before the section of the splanchnic."

The result of these experiments is to show that irritation of the depressor nerves exerts, through the splanchnic, an inhibitory or paralyzing influence upon the abdominal vaso-motor system; and thus, the resistance to the flow of blood being diminished in the immense vascular system of the abdominal organs, the pressure is correspondingly reduced in the arterial trunks.

*Influence of the Pneumogastric upon the Lungs.*—Recent experiments have made important additions to our knowledge of the nervous processes concerned in respiration. In the first place, Flint conclusively shows that the sense of want of air (*besoin de respirer*) is not communicated to the nerve centres by the pneumogastric; for respiration continues after section of those nerves. Farther than this, Brown-Sequard maintains that the generally accepted view that the centre of respiration is located in the medulla oblongata, at the *nœud vital*, or vital point of Fleurens, is erroneous; for, although respiration is instantly arrested by section or a mere prick at this point, yet section of the medulla or the spinal cord below this point, and above the level of the respiratory nerves, does not, in young mammals and adult birds, immediately stop the rhythmical respiratory movements; and he has even found the contractions of the diaphragm to continue for a time, with great regularity, when its communication, not only with the medulla, but also with the spinal cord, has been cut away. From these observations, it is necessary to believe that the diaphragm is, to a certain extent, an independent organ, containing in itself, as does the heart, sympathetic nerve centres controlling its movements. But the vagus, although not indispensable to respiration, yet exerts upon it a powerful influence, whose nature will best be seen by noting the result of certain experiments.

If both pneumogastrics are divided in the neck, in dogs, the number of respiratory acts is at first increased; but, as soon as the animal becomes tranquil, the number is diminished, sometimes falling from sixteen or eighteen to four per minute. The inspirations are, at the same time, unusually profound, attended with excessive dilatation of the thorax. Death usually occurs in from two to five days, and, in most of the animals so dying, the lungs are found engorged with blood, so that they sink in water. This carnification is explained by Bernard as due to a rupture of the pulmonary capillaries, consequent upon a traumatic emphysema, resulting from the excessively deep and labored inspirations.

If galvanization be applied to the pneumogastric in the neck, the effect upon respiration varies, according as the excitation is feeble or energetic. A feeble current accelerates respiration, and renders it more vigorous, especially in inspiration, although, if the feeble current is applied very high up, near the origin of the nerve, respiration is arrested. If a powerful galvanic current is applied to the pneumogastric, in any part of the neck, respiration is arrested by a spasm of the diaphragm; but it speedily returns, even during the excitation. After the removal of the excitation, breathing is accelerated.

Galvanization of the superior laryngeal nerve, whether energetic or feeble, arrests respiration by paralyzing the diaphragm. Galvanization of the recurrent laryngeal generally produces the same effect. Respiration is also sometimes arrested by the galvanic current applied to the pharynx or œsophagus.

Galvanization of the lungs produces persistent spasm of the diaphragm, and consequent arrest of breathing in inspiration.

From these experiments, it appears that the pneumogastrics exert a normal stimulant influence upon the function of respiration, and that, under certain circumstances, they may also exert an inhibitory power. The inhibitory effect is produced in one of two ways:—first, by paralyzation of the inspiratory muscles, induced by irritation of the superior or inferior laryngeal, the pharyngeal or œsophageal branches; and, secondly, by a spasm of these muscles, from irritation of the trunk of the vagus, or its ramifications in the lungs.

The first of these modes of inhibition occurs normally in the act of deglutition. It is impossible to breathe, while swallowing; not only because the larynx is closed by the epiglottis, but also because there is a momentary reflex paralysis of the diaphragm, from irritation of the pharyngeal and superior laryngeal nerves.

In the operation of tracheotomy, a temporary stoppage of respiration not unfrequently occurs, from irritation of the inferior laryngeal nerve. The same effect has been observed by Brown-

Sequard, on suddenly stopping the mouth of a tube introduced into the trachea of dogs or rabbits, all respiratory effort at once ceasing.

Arrest of respiration also takes place upon submersion. That this arrest is not dependent upon the spasmodic closure of the glottis, which also occurs, has been shown by Bean and Brown-Sequard, by submerging an animal in whose trachea a tube had been placed, whose free extremity communicated with the atmosphere. Even in this case, no effort was made to breathe, or if so, not till after some seconds. Bean asks if this arrest is due to the influence of *cold* upon the mouth and nostrils. Brown-Sequard answers the question by submerging an animal in *warm* water, in a similar manner, and finds respiration again suspended. He concludes that the irritation produced by the contact of either cold or warm water upon the mouth and nostrils, produces, by its *suddenness*, a reflex arrest of breathing. If the trunk or limbs are dipped in water, breathing is accelerated; but it is arrested by the sudden dashing of cold water upon any part of the body—an illustration of Bert's law that any gentle irritation of centripetal nerves increases the number of the respiratory movements, and any powerful excitation diminishes them.

The engorgement of the lungs following section of the pneumogastric in the lower animals, is also seen in man, when, from any cause, that nerve is severed. A case occurred at the Boston City Hospital,* in 1865, of a woman, whose husband very skillfully divided with a knife the right pneumogastric, as well as the internal jugular vein and the neighboring muscles. She was attacked, on the tenth day, with congestion of the right lung, which passed off on the thirteenth.

Coughing is a reflex, spasmodic contraction of the expiratory muscles, produced by irritation of the inferior laryngeal or pulmonary branches of the pneumogastric. In asthma, there is, according to Salter, a spasmodic contraction of the bronchial tubes, caused by pneumogastric irritation. This irritation is generally reflex, from impressions upon the respiratory or gastric mucous membrane, or the skin; but may arise, as well, from cerebral disturbance. Neftel † has contributed some interesting observations upon the treatment of this most obstinate affection by galvanization of the pneumogastric. He uses the polar method, applying a current of gradually increasing intensity, all large fluctuations being carefully avoided, by means of the rheostat, and reports a marked degree of success, the paroxysm usually passing off in from two to ten minutes. But he finds the relief afforded sometimes by the anelectrotonic, and at other times by the catelec-

* Boston City Hospital, Medical and Surgical Reports. 1870. Surgical Report by Dr. D. W. Cheever.

† W. B. Neftel. Galvano-Therapeutics. New York. 1871.

trotonic condition of the vagus. Reasoning from the spasm-theory of asthma, it was to be expected that the former alone could give relief; but Neftel has found that in certain cases the attack is arrested by the latter condition, after the former has failed. In explanation of this peculiarity, he suggests that the theory, held by Walshe and others, that asthma is due to a paralytic condition of the muscular coats of the bronchial tubes, may be true of some cases, while others are the result of spasm, the former class being relieved by catelectrotonus and the latter by anelectrotonus. The idea is certainly plausible, but, in the present inchoate condition of galvano-therapeutics, we must await further evidence before accepting it.

(CONCLUDED IN NOVEMBER NO.)

